# Consistent DSC and TGA Methodology as Basis for the Measurement and Comparison of Thermo-Physical Properties of Phase Change Materials

**DOI:** 10.3390/ma13204486

**Published:** 2020-10-10

**Authors:** Lukas Müller, Gabriel Rubio-Pérez, Andreas Bach, Natalia Muñoz-Rujas, Fernando Aguilar, Jörg Worlitschek

**Affiliations:** 1 Competence Centre Thermal Energy Storage (TES), Lucerne University of Applied Sciences and Arts, 6048 Horw, Switzerland; joerg.worlitschek@hslu.ch; 2 Escuela Politécnica Superior, Universidad de Burgos, 09006 Burgos, Spain; grubio@ubu.es (G.R.-P.); nmrujas@ubu.es (N.M.-R.); faguilar@ubu.es (F.A.); 3 Business Unit Materials Characterization, Mettler Toledo GmbH, 8606 Nänikon, Switzerland; andreas.bach@mt.com

**Keywords:** phase change material, latent heat storage, thermal energy storage, differential scanning calorimetry, thermogravimetric analysis, energy

## Abstract

Measuring thermo-physical properties of phase change materials (PCM) in a consistent and reliable manner is essential for system layout of thermal energy storages and correspondingly material selection. Only if basic properties are assessed in a comparable way a selection process leads to the top candidate for any given application and thus enhances market penetration of renewable energy sources coupled with thermal energy storage. In this study, we focus on differential scanning calorimetry (DSC) and thermogravimetric analysis (TGA) as basic assessment techniques and develop consistent measurement procedures to create a database with comparable results. We show consistency of the measured results through analysis of coefficient of variation (CV), being in the mean 1.69%, 0.05%, 0.06% and 4.00% for enthalpy, melting onset, melting peak and maximum operating temperature, respectively. Overall, 23 PCM have been measured with the presented methodology, which was mainly possible due to the reduced measurement and preparation time per PCM compared to standard techniques, while achieving similar accuracy and precision.

## 1. Introduction

The current energy system transformation will lead to an increase of renewable power generation, of which wind and solar photovoltaic are considered to have major contributions. [1] Both technologies require storage concepts due to fluctuations in energy production. Such storage concepts can consist of a manifold of different technologies and types of energy stored. In moderate temperature climate zones, more than half of the energy end-use is governed by space heating [2], therefore, storing energy surplus when available as heat is logical. Furthermore, similar integration concepts can be used for storing cold, which will become a substantial need of the future due to the effects of climate change [2].

One particular technology for short-term heat/cold storage is known as latent thermal energy storage, a system based on a phase change material (PCM), a heat exchanger and a power source. The PCM stores large amounts of energy due to a phase change, commonly liquid-solid, at a specific temperature. In the last two decades, interest in PCM regarding properties, system integration and applications have risen substantially and generated over 4300 research publications [3].

Several different research tracks within the field of PCM development and characterization can be identified. They constitute the modification of PCM through the introduction of additives to overcome undesired properties, for example flammability in organic PCM [4], enhancing the thermal conductivity [5], specific heat capacity [6], or segregation in salt hydrates [7,8]. In addition, the development and integration of bio-based PCM [9,10,11] into several applications such as thermal regulation of buildings [12] or clothing [13].

A recent problem in the development of latent thermal energy storage systems is the variance in published basic thermo-physical properties, particularly melting point and enthalpy. This leads to complicated material selection processes due to uncertainty of the measured data. Therefore, a critical review of several sources or in-house measurements of these basic properties is usually required. The reason for this variance in data lies within the manifold of measurement techniques available and experimental parameters considered, complicating the comparability and selection of materials [14,15,16,17].

Published literature concerning standard measurement methodologies with specific focus on PCM is scarce. There are a number of standards describing differential scanning calorimetry, like DIN 53765 [18], ISO 11357-3 [19], ASTM E 793 [20], and ASTM D 3418 [21], which are either non-specific regarding sample material or for a specific group of materials (e.g. ASTM D 3418 is for polymers). Within those industry standards, the recommended heating rate is usually 10 K min^−1^ for dynamic mode. In PCM publications, several different heating/cooling rates are used, as low as 0.5 up to 10 K min^−1^. [22,23,24,25,26] Barreneche et al. have studied the difference of operating mode, i.e. dynamic or step mode. [27] They found dynamic mode to be suitable for the analysis of organic and inorganic PCM.

It is common in published literature to omit important data used during a study. The sample mass during an investigation is usually not reported in PCM studies. Castellón et al. have shown that the sample mass has an influence on the properties as well as the heating rate [28]. A standard for the measurement of properties of PCM was published by the RAL Institute in Germany, and a round robin test was carried out in the context of Task 42 from the IEA Solar Heating and Cooling Program [29,30,31]. These publications cover a wide range of elements that need to be taken into account, like influence of sample mass, heating/cooling rate or type of analysis. However, a detailed standard methodology that could be used as a research model in the laboratory is still lacking. Additionally, the preparation and measurement of this standard uses differential scanning calorimetry (DSC) measurements exclusively, resulting in a lack of variability of measured PCM. Furthermore, the change in heating/cooling rate per measured substance results in a limited comparability of properties.

Regarding thermogravimetric analysis (TGA) methodology, an even greater disagreement is found. Experimental parameters vary widely, the reported results are manifold, and therefore a comparison between different PCM is difficult to impossible. Some standards describing the thermogravimetric analysis do exist, for example, ASTM C 1872 [32], ISO 11358-1 [33] and ASTM E 1131 [34]. The first two are specific to a certain material group, while the latter is rather generic and vague. None of the above-mentioned standards suggest a method for analysis of PCM specific properties, thus we present a possible evaluation of the maximum operating temperature within this work.

In this work, we present two measurement protocols applicable to a variety of different materials, all of them used as PCM with melting points between −50 °C and 190 °C. Using consistent experimental parameters, we demonstrate a comparison of the resulting material properties for an accurate evaluation of application specific PCM. We present a set of PCM materials, their thermal characteristics and compare the data with presented literature data. These measurement protocols are the basis for the development of an open-source PCM database, which does not currently exist.

## 2. Materials and Methods

### 2.1. Materials

The materials discussed and measured in this work are listed in Table 1. A variety of PCM classes have been investigated, including organic and inorganic PCM. Selection has been based on testing different PCM classes, including at least one per material class and melting point focusing on two aspects. First, temperatures relevant for building applications (15 °C, 30 °C and 60 °C), and second, covering the temperatures from −50 °C up to 190 °C with several materials. Additionally, the criteria of commercial availability were taken into account. Furthermore, the measurement methodology is applied to both commercial PCM, as well as chemical substances.

### 2.2. Equipment

The investigated materials have either been tested as delivered, or, if necessary, were mixed and stirred at a temperature above their melting point for at least 30 min. The following devices have been used for DSC measurements: The Mettler Toledo DSC 823e (Nänikon, Switzerland) with FRS 5+ sensor (Mettler Toledo, Nänikon, Switzerland) and TC100 Intracooler (Huber Kältemaschinenbau AG, Küsnacht, Switzerland), the Mettler Toledo DSC 3+ (Nänikon, Switzerland) with FRS 6+ sensors (Mettler Toledo, Nänikon, Switzerland) and both TC45/100 Intracoolers (Huber Kältemaschinenbau AG, Küsnacht, Switzerland). For the TGA measurements, a TGA/DSC 2, TGA/DSC 3+ and TGA/DSC 1 from Mettler Toledo all equipped with a DTA or DSC sensor were used. Equipment is shown in Supplementary Figure S1.

### 2.3. DSC Measurement Methodology

#### 2.3.1. Preliminary Steps

- The DSC was turned on approximately an hour before the measurement to allow the furnace to reach thermal equilibrium.
- Purge gas flow has been set to 20 mL min^−1^, and protective gas always 10 mL min^−1^ higher to avoid the entrance of sample vapors in the inner parts of the DSC. Nitrogen (N_2_) has been used.
- The DSC has been checked regularly, at least once per month, and a calibration using Indium (In) prior every series of measurements was performed.
- Gloves and tweezers have been used for sample handling to avoid any contamination of the sample and the furnace.

#### 2.3.2. Sample Preparation

A suitable sample crucible has been chosen considering compatibility with the measured PCM. Aluminum crucibles were mostly used, but if degradation or reaction of the PCM with the material of the crucible occurs, alumina crucibles could be considered.

When preparing the sample, a representative portion from the bulk material was chosen. If needed, it was mixed or stirred properly before preparing the sample.

If the sample was liquid, a syringe or a disposable pipette (made of glass or plastic) was used to pour the sample into the crucible. If the sample was solid, a micro spatula was used. The bottom of the pan was always fully covered when pouring the sample, to ensure proper heat transfer. Concerning amounts of substance in the crucible, 5 μL were used when the sample is liquid and 5 to 10 mg when solid. Three examples are shown in Supplementary Figure S2.

Both the mass of the empty pan and the poured sample have been measured using a scale with a maximum tolerance of ± 0.001 mg.

Any thermal alternation or contamination of the sample was avoided during sample preparation to avoid untrue results.

#### 2.3.3. Temperature Program

**Figure 1:**
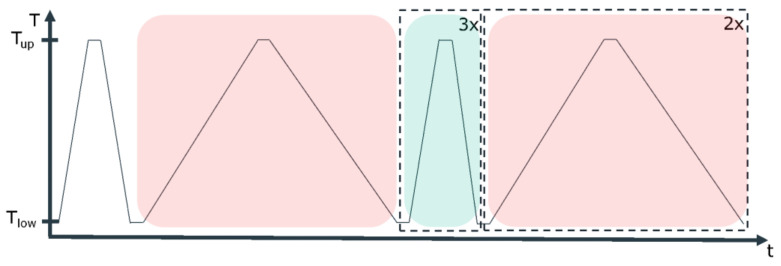
Complete temperature program for the differential scanning calorimetry (DSC) measurements. The upper and lower temperature limits, T_up_ and T_low_, are indicated, as well as the two heating rates applied in the experiment. The dashed boxes indicate the number of repetitions per heating rate, while the colored boxes indicate the segments used for analysis.

The temperature program consists of several cycles at two distinct heating/cooling rates, as shown in Figure 1.

The faster heating rate is set to 10 K min^−1^, and the slower to 2 K min^−1^. The purpose of the first cycle is to delete the thermal history of the sample before the measurement. The other six cycles will be used to determine the phase change enthalpy and temperature, as will be explained later.

The upper and lower limits, T_up_ and T_low_, are different for each PCM, as they are set with respect to its melting point, T_m_, according to the following rule:

- T_up_ = T_m_ + 30 K
- T_low_ = T_m_ − 20 K

All the isothermal segments in between dynamic steps have a duration of 2 min to ensure thermal stabilization.

#### 2.3.4. Measurement

In order to obtain a better statistical distribution of the results, at least 3 individual samples for each PCM were measured.

The crucibles have been checked by measuring the weight of the sample after the measurement to ascertain the sample is intact.

#### 2.3.5. Data analysis

**Figure 2:**
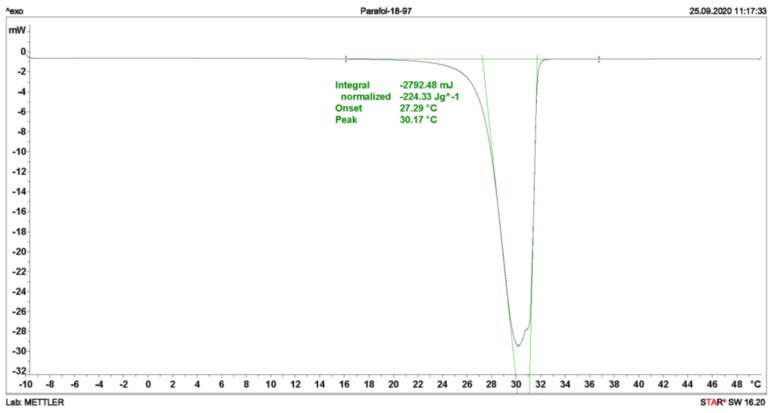
Graphical example of the determination of enthalpy, the integral of the signal with respect to the baseline before and after the peak, onset, as intersection between baseline and green tangential on the left, and peak, as highest point of the peak.

The last three segments at 10 K min^−1^ heating rate (indicated by green in Figure 1) are used to determine the phase change enthalpy, calculating the average and the standard deviation. The enthalpy is considered to be the area under the peak (see Figure 2), thus, the integral value of the differential heat flow in accordance with ASTM E 793 [20]. The use of the first derivative of the DSC curve was used to find more precisely both the start and the end of the melting, i.e. the points where the first derivative is zero for a certain length.

The three segments at 2 K min^−1^ heating rate (indicated by red in Figure 1) have been used to determine the phase change temperature, calculating the average and the standard deviation. The onset temperature is considered to be the phase change temperature of the PCM, and it is obtained according to ASTM E 2253 [35]. Additionally, the peak temperature defined as maximum value per signal is also determined (see Figure 2).

The information provided by the cooling segments has been used to study the sub-cooling tendency of the PCM qualitatively. However, the DSC is not a suitable instrument to determine sub-cooling behavior for applied scale PCM.

### 2.4. TGA Measurement Methodology

#### 2.4.1. Preliminary Steps

The TGA was turned on the night prior to the measurement, such that the cryostat and the balance could stabilize properly overnight.

As long as the TGA is on, both protective and purge gas were allowed to flow through the device. Nitrogen (N_2_) was used for common measurements. Oxygen (O_2_) or air could be used as purge gas if oxidation or combustion of the sample are intended, but those measurements are not presented within this work. Protective gas flow was around 30 mL min^−1^, and purge gas flow always 10 mL min^−1^ lower to avoid the entrance of vapors from the sample into the inner parts of the TGA.

The TGA has been checked regularly, at least once per month, and a valid calibration was performed at least once per year. 

Tweezers and gloves have been used in every operation, as neither the crucibles nor the inner parts of the furnace should be touched with bare hands to avoid contamination of the sample or the furnace.

#### 2.4.2. Blank Measurement

To correct the buoyancy effect, blank measurements were carried out before every set of measurements. An empty crucible was placed in the balance and a measurement launched with the same exact conditions (heating rate, gas flow, kind of crucible, etc.) as the ones expected to be used in the measurement of the sample.

If the TGA has not been used for a long period of time, three blank measurements have been performed. The first one was discarded and the most stable curve from the other two was chosen. A new blank measurement was launched every four samples (or every 5 h) approximately, to ensure the use of a representative blank line. A new blank measurement was launched every time the conditions of the experiment changed (e.g. temperature range, gas flow or heating rate).

#### 2.4.3. Sample Preparation

- The material of the crucible has to be compatible with the substances measured. Alumina crucibles have been used for all measurements.
- The sample should be representative of the bulk material, so it was stirred or mixed properly before extraction of the sample.
- A syringe or a disposable pipette was used when handling liquid samples. Solid samples were ground with a mortar, generating a powder as fine as possible. A micro spatula was then used to pour the sample into the crucible. The bottom of the crucibles should be fully covered. If grounding the PCM was not possible, samples with the same shape and size have been prepared to ensure repeatability.
- The amount of material poured on each crucible depends on the nature of the PCM: 5 to 10 mg for organic PCM and 10 to 30 mg for inorganic PCM, accounting for the general difference in density of these material categories, thus ensuring the coverage of the crucible bottom.
- The sample mass has been measured using a scale with a maximum tolerance of ± 0.001 mg. This mass has also been checked by the balance of the TGA itself.
- Caution was required to not alter the samples thermally before measurement, in order to avoid untrue results.

#### 2.4.4. Temperature Program

When defining the temperature program of the experiment, the following steps are performed:

- An isothermal step of 10 min at 40 °C, to ensure thermal stability of the sample before the heating step.
- A dynamic step from 40 °C to 600 °C at a constant heating rate of 10 K min^−1^.
- If a DSC/TGA has been used and the DSC curve is needed, an isothermal step of 10 min at 600 °C to correct the heat flow curve drift if necessary.

**Figure 3:**
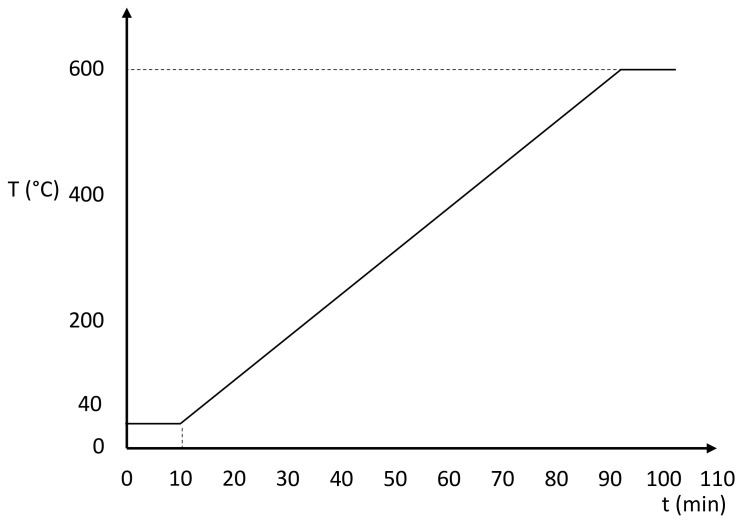
Complete temperature program for the thermogravimetric analysis (TGA) measurements. Duration and temperature of each step within the program are indicated, applied to all materials.

A diagram of the complete temperature program is represented in Figure 3.

The upper limit of 600 °C has been found to be enough for the PCM measured within this work.

#### 2.4.5. Measurement

Three different samples from each PCM have been prepared and measured, in order to obtain a better statistical distribution. If the alumina crucibles were reused, they were cleaned with a mixture 0.1M of hydrochloric acid (HCl) in water, put in a muffle oven and kept at high temperatures for some time, to ensure that all the residues have been removed. When crucibles were too deteriorated, they were discarded.

#### 2.4.6. Data Analysis

Before starting the analysis, the most stable baseline from the performed blank measurements was chosen and subtracted from the TGA curve of the measured PCM. To account for any thermal event reliably, the first derivative of the mass loss curve, DTG, was calculated.

**Figure 4:**
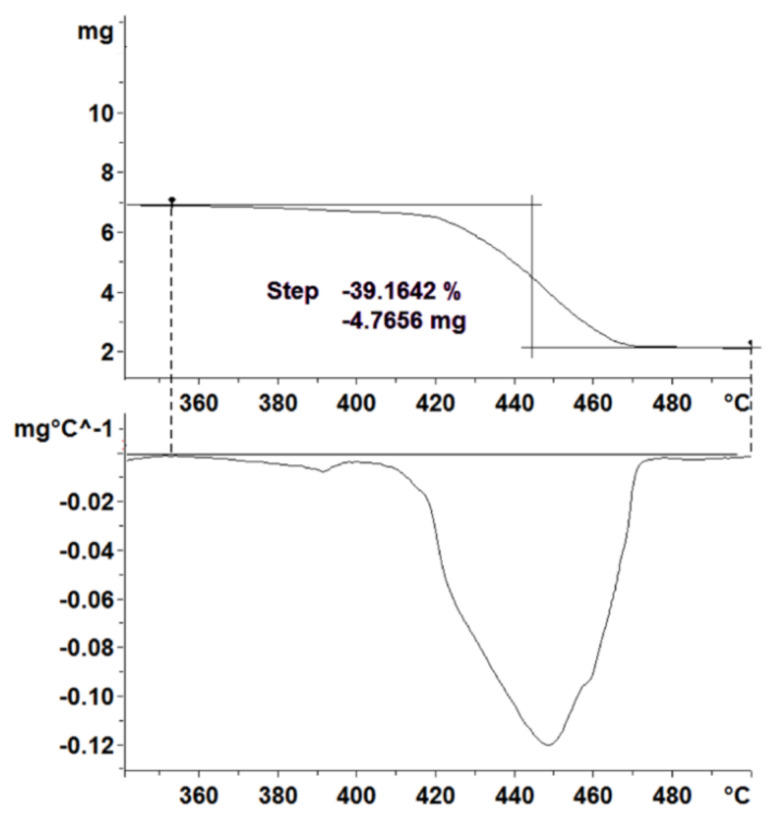
Calculation of the mass loss as relative change in mass of an event in a TGA curve. The beginning and end of a thermal event are determined using the first derivative curve (DTG) curve as shown in the lower graph.

The mass loss of a specific event was determined by tracing two horizontal lines over the TGA curve, one before the event and one after it, and taking the difference of these two horizontal values. The DTG curve was used to locate both the start and the end of the event, that is, the points in which the value of the curve is zero. This can be seen in Figure 4.

When several samples of the same material were compared, the relative mass loss curve has been used instead of the absolute one, to discard the effect of having different sample masses and being able to make a proper comparison.

If the TGA curve shows several steps, such as the TGA curve of a salt hydrate, each one of them has been analyzed as an independent event with its own onset temperature and mass loss.

**Figure 5:**
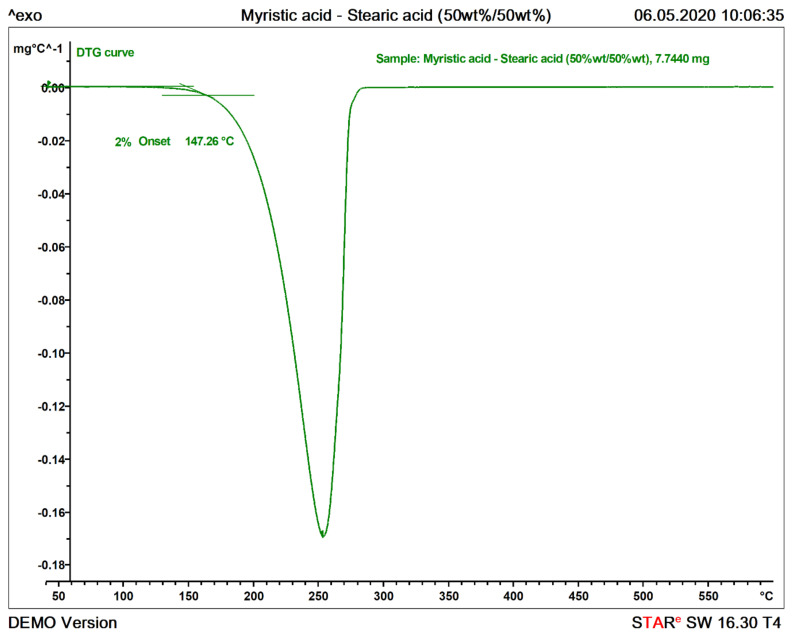
Calculation of the maximum operating temperature on a DTG curve. Maximum operating temperature is defined as the intersection of the tangential line on stable condition (left bound) and the tangential line at 2 % change in mass loss rate (right bound).

We define the maximum application temperature of the PCM from the TGA experiments by considering the 2% onset temperature of thermal decomposition of the sample, calculated by evaluation of the first derivative curve (DTG), as shown in Figure 5. The 2% threshold value is relative with respect to the peak height between the start temperature at 40°C and the first peak minimum on the DTG curve. The tangents of the evaluation and the horizontal line marking the 2% threshold are also shown in Figure 5. This methodology is based on analogue evaluations followed for other materials, such as in the definition of the glass transition temperature described in the standard DIN 65583 [36]. Following these steps, a quantitative analysis of the TGA curve was obtained.

## 3. Results and Discussion

### 3.1. DSC Measurements

The following paragraphs will demonstrate the precision and accuracy of the method introduced in Section 2.3 for two different PCMs as an example. Furthermore, the extension to other PCM classes will be discussed on several examples from different classes each showing certain characteristics in the heat flux curve.

#### 3.1.1. Validation

**Figure 6:**
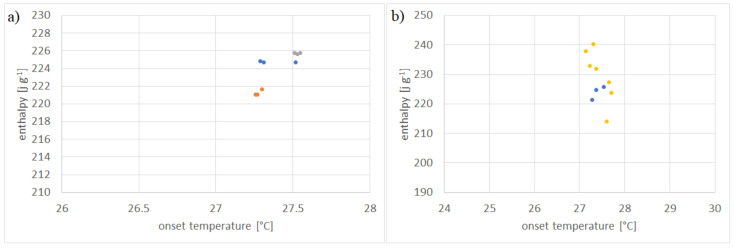
(**a**) Experimental results of enthalpy over onset temperature of Parafol 18-97 (octadecane) measured three times, as per measurement protocol suggested in Section 2.3. Each color depicts one measurement run. (**b**) Comparison of mean enthalpy and onset temperature from measurements (blue) with published data on standard DSC technique (yellow) [37].

The proposed measurement methodology was validated using Parafol 18-97 (octadecane). This PCM has been measured and evaluated previously in a round robin experiment by Gschwander et al. [36]. The herein proposed measurement protocol has been repeated for 3 times, each indicated in Figure 6a by a different color. The measurements have shown that, throughout an experiment, the coefficient of variation (CV) for enthalpy and onset temperature is between 0.02–0.12% and 0.06–0.38%, respectively. This shows the high precision of the protocol. All those measured are shown in Supplementary Table S1.

In Figure 6b, the mean of each experiment is compared to the results of the round robin test performed by Gschwander et al. [37]. It is evident that the proposed protocol is as accurate as the methodology proposed by the aforementioned authors. The CVs for all measurements performed by them for enthalpy and onset temperature are 3.87% and 0.8%, respectively. Including the results from our experiments, they become 3.71% and 0.75%, indicating that the proposed methodology has at least the same accuracy.

**Figure 7:**
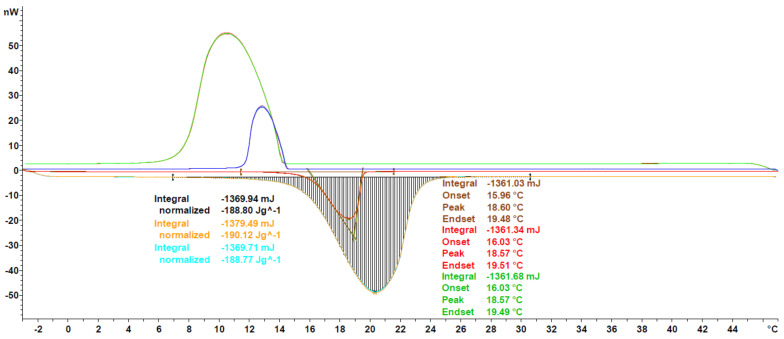
Heat flux curve of Crodatherm17 for a single experiment. Heating (lower) and cooling (upper) segments are shown, though only heating segments are evaluated for enthalpy and onset, peak and endset temperatures, as described in Section 2.3.5.

In order to further validate the proposed measurement protocol, a small round robin test between the labs of Mettler Toledo and Hochschule Luzern and four different measurement devices (see Section 2.2) has been performed. The experiments were performed with Crodatherm 17 (see Table 2) from the same production lot and repeated between 4 and 8 times per device. A typical measurement curve and the results of the data analysis, as per Section 2.3.5, can be seen in Figure 7. Measured heat flux throughout the experiments is highly consistent, as indicated by the low CV values for enthalpy and onset temperature, being 0.41% and 0.25%, respectively. Furthermore, the cooling curves are also shown as qualitative evaluation. Since Crodatherm17 is an organic PCM, it has low sub cooling, which is typical for this class of PCM [9,38].

The mean results of each measurement series and the overall average are shown in Table 2. The calculated CV for enthalpy is in the same range as for the Parafol 18-97, indicating the robustness of the measurement protocol for other PCM classes. On the contrary, the CV for the onset temperature shows greater variance in different labs with a value of 1.73%, which is almost double what has been reported for Parafol 18-97. Nevertheless, the value remains low and, having a closer look into mean and standard deviation, the measurement procedure can still be regarded as accurate.

To sum up the findings of the comparison between different methodologies and different laboratories, it should be pointed out that precision and accuracy are kept on a high quality level indicated by the small CV being 3.71% and 0.75%, and thus smaller when including our results into the round robin results from [37]. Furthermore, the CV of measurements performed in different laboratories is in the same order of magnitude for enthalpy. Variations of the onset temperature are higher, but are regarded as small with respect to the absolute values.

An advantage of the methodology is the time needed to execute the measurement of a PCM. Compared to the RAL methodology [29], the DSC characterization presented in this work delivers results within approximately three days, while the RAL includes measurements of approximately seven days’ duration. This leads to higher cost and thus to a lower number of PCM measured.

#### 3.1.2. Extension to Different PCM Classes

To show the suitability of the method for other PCM classes, a few example measurements are discussed in detail, along with its comparison with literature values and a description of typical effects encountered during measurements.

The first example is another organic PCM, octadecanoic acid, one of the most common saturated fatty acids [39]. Published values of enthalpy and melting temperature can vary largely, to an extent of 60 J g^−1^ and 18 °C, respectively, as can be found on the National Institute of Standars and Technology (NIST) chemistry webbook, SRD 69 [40,41]. The values reported in this work are 230.81 J g^−1^ and 67.65 °C, which is in good agreement with the majority of the NIST values, having a relative deviation of less than 5%.

**Figure 8:**
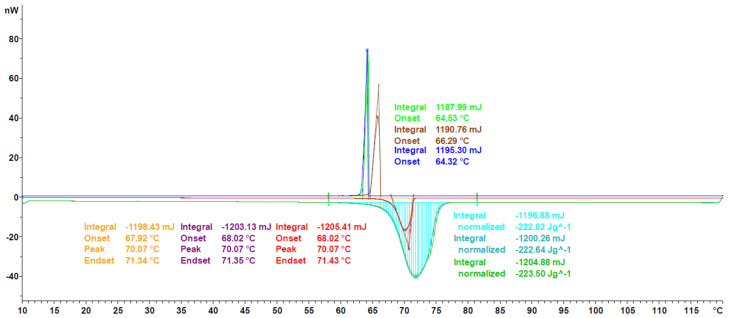
Heat flux curve of octadecanoic acid for a single experiment following the evaluation as per Section 2.3.5 is shown.

As can be seen in Figure 8, the generic melting/solidification behavior is similar to the ones of Crodatherm 17, though a difference in onset temperature for the cooling curves can be observed. This difference is an indicator of the validity of the classical nucleation theory and has been observed for a variety of other PCMs as well [42]. Due to the size-dependence through the number of possible nucleation sites, the effect of sub or even super cooling is more pronounced in DSC measurements, than it would be in applications. The temperature range of onset temperatures during solidification is given as a qualitative estimation of the tendency to supercool. Nevertheless, it must be mentioned here that the true amount of sub or super cooling has to be addressed and measured for each application in the corresponding sample size.

**Figure 9:**
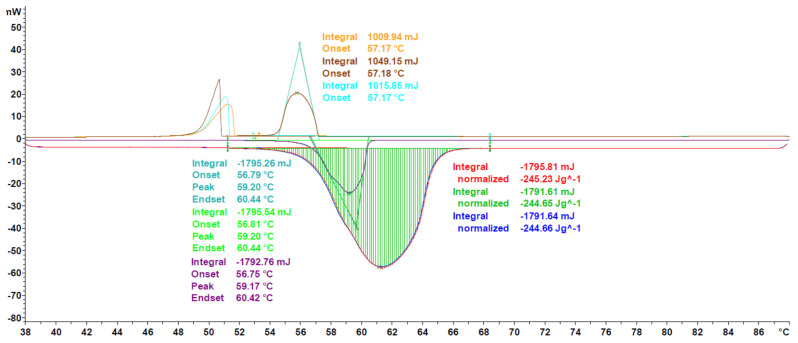
Heat flux curve of 1-octadecanol for a single experiment following the evaluation as per Section 2.3.5 is shown.

A further characteristic of organic PCM, polymorphism, can be seen for example in the measurement of 1-octadecanol, a fatty alcohol. As can be seen in Figure 9, two distinct peaks are visible in the cooling curves, indicating the formation of two different polymorphs. The confirmation for this observation is given through the existence of a slight shoulder on the left side of the measurement peaks of the heating curves. This phenomenon has already been described in literature by Ventolà et al. where also the thermo-physical properties are consistent with the ones measured in this work [43].

Additional thermo-physical data on 1-octadecanol can also be found in NIST chemistry webbook, SRD 69 [40]. The values stated for enthalpy have great variance, resulting in a CV of 30%, while as the CV for melting temperature is at just 3%. The results obtained in this work, 247.6 J g^−1^ and 56.61 °C, are in good agreement with two of the four NIST stated enthalpy values, with a relative difference of less than 5%, but between 0.6% and 8% off for stated temperatures. This offset could be due to the use of different experimental procedures, as results from other techniques apart from DSC are also published by NIST. Furthermore, it remains unclear if the onset temperature or the peak temperature has been selected as the phase change temperature.

**Figure 10:**
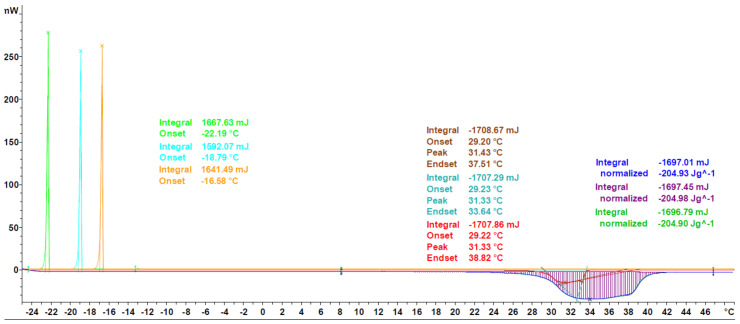
Heat flux curve of calcium chloride hexahydrate for a single experiment, following the evaluation as per Section 2.3.5, is shown.

Calcium chloride hexahydrate, a salt hydrate, is shown in Figure 10, as an example of inorganic PCM. The main difference in the characteristic of inorganic PCM compared to organics is the highly pronounced super cooling effect, as has been described in literature [44]. Taking into account the mass dependence of the effect (as has already been mentioned), as well as the usually higher densities of salt hydrates (two to three times the one of organic PCM), the methodology has been adjusted to employ a greater mass (up to 40 mg) in the measurements of salt hydrates. Furthermore, the isothermal segments between cycles can be prolonged, in order to guarantee crystallization of the sample before the next heating segment.

Regarding the thermo-physical data, the value measured in this work is in good agreement with values reported by Schmit et al., for several manufactures, having a deviation of less than 3% for the mean value of enthalpy [45]. As shown in Figure 10, a single measurement can have a higher deviation, in the range of 5%, but precision and accuracy can be optimized through the execution of at least three individual experiments with three evaluated segments. Furthermore, it has to be taken into account through the work of Schmit et al., a heating/cooling rate of 2 K min^−1^ is used to measure all thermo-physical data, whereas in this work, a heating rate of 10 K min^−1^ is used to determine enthalpy, which could lead to differences in the recorded values.

### 3.2. TGA Measurements

The proposed TGA methodology has also been validated through a series of measurements. The PCMs studied in this work have been measured in the same laboratories using a TGA 2 from Mettler Toledo.

Three samples of each PCM have been measured, and both the relative mass loss of each step observed in the TGA curve and the maximum operating temperature were extracted from the results, following the aforementioned definitions.

The relative mass loss of each step of some of the PCM has been studied, as well as their mean value, standard deviations and CV, which are shown in Table 3 as an example. It shows that the coefficient of variation is low in all of them (lower than 1%). This is a good indicator of the precision of the proposed measurement methodology.

Concerning the maximum operating temperature, the 2% onset temperature in the first derivative of the TGA curve criteria is proposed, due to similarities with the definition of the glass transition temperature in DMA experiments, where a 2% threshold in the curve is also specified. Further information can be found in the standard DIN 65583 [36]. The CV of the maximum operating temperature of the results has also been calculated, having a mean value of around 4% considering different PCM and devices, another good indicator of the quality of the procedure.

**Figure 11:**
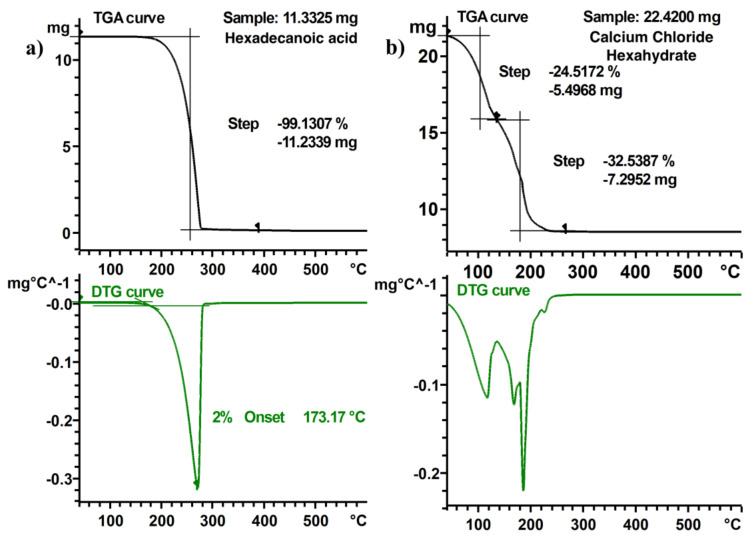
TGA and DTG curves of (**a**) hexadecanoic acid and (**b**) calcium chloride hexahydrate.

Examples of TGA and DTG curves with the extracted results are shown in Figure 11. Hexadecanoic acid experiments decompose in one single step, but two steps can be observed in the curve of calcium chloride hexahydrate, due to the dehydration. No lids were used with the crucibles in the measurements; so, in this case, water loss starts at a temperature below 40 °C. This is the reason why no maximum operating temperature is displayed because, in this case, the proposed definition loses its meaning.

Comparison of the results with literature is difficult, as a new definition of maximum operating temperature is proposed. However, some other PCM have been measured to compare the experimental results with previously published data. The onset temperature and the temperature in which the mass loss rate is the maximum of three PCM have been compared with the results provided by several authors. The deviation of the results with each one of the papers is shown in Table 4. It can be seen that, even for the same PCM and for the same property, different authors report different results. Furthermore, the same paper states that a high deviation for the onset temperature shows high deviation and a low deviation for the temperature of the maximum mass loss rate. This is most likely due to differences in the definitions of the measured properties. This qualitative comparison can only be considered as a proof of the need of a common methodology in the measurement of PCM properties.

In summary, it can be stated that measured mass loss curves show very small deviations per PCM, thus indicating the consistency of the methodology. The comparison with literature for TGA curves is rather difficult, due to the large deviations in methodology, and stresses the need for a precise methodology to allow for comparison in between PCM.

### 3.3. Database Measurements

A total amount of 23 PCM from 9 different classes have been characterized through DSC/TGA, using the same measurement protocol as described in Section 2. An extract of the most important properties (i.e. melting enthalpy and onset, peak, crystallization onset and maximum operating temperature) and indication of standard deviation can be found in Table 5. Measuring such a large amount of different PCM is only possible due to the low cost of the methodologies presented, i.e. the smaller amount of time needed for measurements.

The PCMs have been selected using several criteria. Firstly, a manifold of 9 different PCM classes was selected to enable a quantitative comparison between classes for any specific application. Furthermore, most applications require a specific temperature range from the storage material, so PCMs with similar meting/crystallization temperatures were selected. For example, PCMs with phase change temperatures around 30 °C or 60 °C are of importance in the building sector, namely residential heating and domestic hot water [51].

Additional PCMs were chosen to show the versatility of the method over a wide temperature range; from −50 to 186 °C. Only some TGA measurements revealed a loss of material within the isothermal segment at the beginning, which indicates a maximum operating temperature below 40 °C. Within Table 5, PCMs exhibiting such a mass loss are indicated with “not applicable” (n/a), since an evaluation with the methodology proposed is not meaningful.

**Figure 12:**
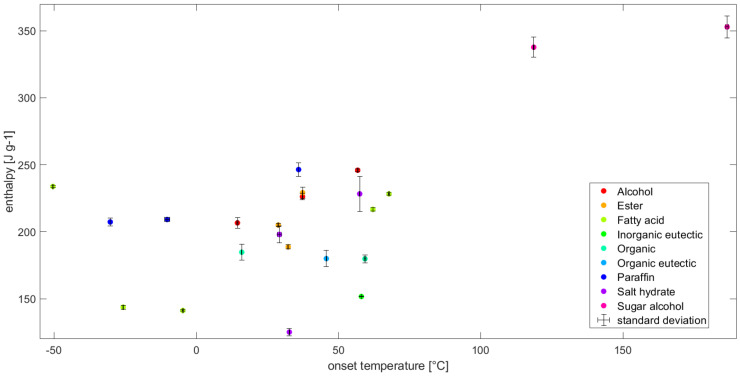
Enthalpy over onset temperature of materials, as given in Table 5. Additionally, the standard deviation is indicated by error bars showing their narrow distribution around the mean.

Figure 12 shows the two most important properties, enthalpy over onset temperature for the PCM given in Table 5. One can see that that the values for enthalpy are distributed in between 120 and 250 J g^−1^. The consistency of the methodology allows for comparison and correlation between different PCMs, and even between PCM classes. It has to be mentioned though, that the number of data points is too small to draw any conclusions about the nature of the distribution, but there seems to be a uniform distribution over all temperatures below 100 °C. It would require a systematic analysis per class, such as for example, the one conducted by Ravotti et al. [9]. The only PCMs measured above 100 °C show substantially higher enthalpy, with values around 350 J g^−1^. Both of them are sugar alcohols which are known to have higher enthalpy values than other PCM, but are only available with melting points around 100°C and above [52,53,54]. These properties correspond well with what is reported in literature for PCM classes [55].

## 4. Conclusions and Outlook

It is shown in this work that a consistent DSC and TGA methodology can be used to consistently measure and compare thermo-physical PCM properties. This facilitates the selection process for a specific application, and guarantees true conclusions, which could not necessarily be drawn from a literature review, due to the reported differences in measurement protocols. Furthermore, a new definition of maximum operating temperature has been introduced, which accounts for first changes in mass upon temperature exposure, and could indicate irreversible degradation processes, regardless of PCM class.

The choice of two different heating/cooling rates became obvious to guarantee precision and accuracy for the two most relevant thermo-physical properties, enthalpy and onset temperature. Enthalpy measurement has better accuracy for higher heating/cooling rates, while the higher heating rate significantly influences the onset temperature. Thus, a second heating/cooling rate was introduced in order to assess the onset temperature in the same experiment. Additionally, the repetition of cycles allows for having statistics within one experiment and additionally shows consistency of phase change, which is essential for almost all applications.

When extending the measurement procedure to different PCM classes, it became apparent that minor changes have to be introduced for certain materials. For the DSC protocol, it was shown that materials with a super cooling tendency need an adjustment in order to observe crystallization events and thus subsequent melting of the PCM, in order to be able to evaluate the thermo-physical properties. The adjustments consist of decreasing the lower temperature limit, increasing the time of isothermal segments in between cycles and allowing for higher sample masses. The first two adjustments do not significantly influence the evaluation, since the heating/cooling rates are kept constant. The weight adjustment can have a minor influence on both properties, but is needed to create crystallization events. Furthermore, it must be mentioned that an increase in mass is generally allowed for PCM with higher densities, thus volume is kept similar.

The presented methodology has some limitations, which include super cooling, size, starting temperature and encapsulation, leading to a possible lack of measured values. The super cooling limits the application of the DSC methodology, such that a stable super cooling will not lead to recrystallization and thus no detection of further melting events throughout the procedure. This could be partially omitted by increasing the holding times at the lower temperature. Limitation of size for DSC methodology has been introduced and further enhances super cooling tendency. Therefore, caution has to be taken by reporting the super-cooling temperature, which can largely deviate from application specific size of sample. A large limitation of the TGA methodology is the starting temperature at 40 °C to reach thermal equilibrium, which is given through the instrument used. This limitation leads to unavailable data for PCM, where evaporation starts within the isothermal segment. Lastly, it needs to be stated that the results obtained are for unmodified PCM, except commercially available ones measured, and thus, the results do not include any change in properties through addition of nucleators, thickeners or any kind of encapsulation.

It is worth mentioning that the data published in this work will be included in an open-source PCM database and into a so-called reference library of the StarE software.

In conclusion, the measurement procedure presented in this work can be successfully applied to a manifold of PCM classes delivering a concise set of thermo-physical properties. This is the basis to develop an open-source PCM database with comparable and reliable data, in order to facilitate the market penetration of PCM based products.

Further research will be directed to include further important properties for a thorough selection process, such as specific heat capacity, thermal conductivity and density. The corresponding measurement methodologies require standardization, and thus comparative studies with appropriate design. Additionally, the extension of the presented methodology to encapsulated PCM could be addressed and validated. At last, the measurement of further PCM with the presented methodology is regarded to be the most important point, in order to enable correlations and support a sound material selection. A strong emphasis on the inclusion of materials not yet characterized is suggested.

## Figures and Tables

**Table 1:** Materials investigated within this study and the corresponding purity, manufacturer and classification.

PCM	CAS Number	Purity	Manufacturer	PCM Class
1-Octadecanol	112-92-5	99%	Sigma-Aldrich	Alcohol
1-Tetradecanol	112-72-1	97%	Sigma-Aldrich	Alcohol
1-Undecanol	112-42-5	99%	Sigma-Aldrich	Alcohol
Ethyl octadecanoate	111-61-5	97%	Sigma-Aldrich	Ester
Methyl hexadecanoate	112-39-0	99%	Sigma-Aldrich	Ester
Methyl octadecanoate	112-61-8	96%	Sigma-Aldrich	Ester
(MgCl_2_-Mg(NO_3_)_2_)·6H_2_O (41 wt%/59 wt%)	7786-30-3/13446-18-9	98%/99%	Sigma-Aldrich	Eutectic
Myristic acid-Stearic acid (50 wt%/50 wt%)	544-63-8/57-11-4	99%/98%	Sigma-Aldrich/Roth	Eutectic
Hexadecanoic acid	57-10-3	99%	Sigma-Aldrich	Fatty acid
Hexanoic acid	142-62-1	99%	Sigma-Aldrich	Fatty acid
Methanoic acid	64-18-6	96%	Sigma-Aldrich	Fatty acid
Octadecanoic acid	57-11-4	95%	Sigma-Aldrich	Fatty acid
Propionic acid	79-09-4	99.50%	Sigma-Aldrich	Fatty acid
Crodatherm 17	n/a ^1^	As purchased ^1^	Croda Europe	Organic
PEG 10000	25322-68-3	n/a	Sigma-Aldrich	Organic
Decane	124-18-5	99%	Sigma-Aldrich	Paraffin
Dodecane	112-40-3	99%	Sigma-Aldrich	Paraffin
Eicosane	112-95-8	99%	Sigma-Aldrich	Paraffin
CaBr_2_·6H_2_O	71626-99-8	98%	Sigma-Aldrich	Salt hydrate
CaCl_2_·6H_2_O	7774-34-7	98%	Sigma-Aldrich	Salt hydrate
SAT	n/a ^1^	As purchased ^1^	Cowa Thermal Solutions	Salt hydrate
Dulcitol	608-66-2	99%	Sigma-Aldrich	Sugar alcohol
meso-Erythritol	149-32-6	99%	Sigma-Aldrich	Sugar alcohol

^1^ On commercial PCM, no data regarding CAS number, purity nor composition are given by the manufacturers. Any data not available is indicated by n/a.

**Table 2:** Comparability of method used by different operators and instruments for Crodatherm 17/DEV 2067. On each instrument, at least four and up to eight runs of the procedure described in Section 2.2 are considered. The mean ± standard deviation and the coefficient of variation are given for each set of measurements.

Laboratory	Operator	Instrument ^1^	Enthalpy (J/g)	CV (%)	Onset Temperature (°C)	CV (%)
HSLU ^2^	1	A	165.32 ± 0.63	0.38	15.84 ± 0.06	0.37
HSLU ^2^	1	B	156.41 ± 0.72	0.46	16.48 ± 0.04	0.26
MT ^3^	2	C	171.15 ± 0.61	0.35	16.08 ± 0.21	1.3
MT ^3^	2	D	169.32 ± 0.71	0.42	15.77 ± 0.03	0.19
Mean	-	-	165.55 ± 6.56	3.43	16.05 ± 0.32	1.73

^1^ A: Mettler Toledo DSC 823e, FRS 5+, TC100 Intracooler; B–D: Mettler Toledo DSC 3+, FRS 6+, TC45/100 Intracooler. ^2^ Hochschule Luzern. ^3^ Mettler Toledo.

**Table 3:** Relative mass loss in decomposition, mean, standard deviation and CV of some of the studied PCM.

PCM	Mass Loss (%)	Mean (%)	Standard Deviation (%)	CV (%)
1-Tetradecanol	99.95	99.93	0.02	0.02
	99.92			
	99.92			
Methyl Hexadecanoate	98.73	98.38	0.34	0.35
	98.04			
	98.39			
PEG 10.000	97.63	97.66	0.16	0.16
	97.84			
	97.52			
SAT	32.76	33.09	0.31	0.93
	33.36			
	33.15			

**Table 4:** Mean values of the deviation of experimental results compared with the corresponding literature.

Reference	PCM	Deviation Onset Temperature (%)	Deviation Temperature Maxmium Mass Loss Rate (%)
Xian Van et al. [46]	PEG 6000	-	−3.36
Zhi Chen et al. [47]	Stearic acid	141.82	11.10
Yaxue Lin et al. [48]	Stearic acid	45.53	0.61
Fang Tang et al. [25]	Octadecane	-	−15.62
Hao Wang et al. [49]	Octadecane	26.94	-
Chaoen Li et al. [50]	Octadecane	35.40	-

**Table 5:** Overview of the properties for all the PCM measured in this work. Given are the mean ± standard deviation or the range of crystallization onset temperature as minimum and maximum measured interval.

PCM	CAS	PCM Class	Melting Enthalpy (J/g)	Melting Onset (°C)	Melting Peak (°C)	Crystallization Onset (°C)	Max. Operating Temperature (°C)
Methanoic acid	64-18-6	Fatty Acid	233.81 ± 0.95	−50.4 ± 0.02	−49.4 ± 0.01	−38.74–−33.74	n/a ^1^
Decane	124-18-5	Paraffin	207.32 ± 2.99	−30.2 ± 0.02	−27.8 ± 0.24	−40.33–−36.97	n/a ^1^
Propionic acid	79-09-4	Fatty Acid	143.7 ± 1.44	−25.7 ± 0.15	−22.2 ± 0.08	−46.6–−44.49	n/a ^1^
Dodecane	112-40-3	Paraffin	209.14 ± 1.45	−10.2 ± 0.94	−8 ± 0.25	−14.69–−13.45	n/a ^1^
Hexanoic acid	142-62-1	Fatty Acid	141.21 ± 0.58	−4.7 ± 0.2	−1.2 ± 0.06	−14.59–−12.09	55.4 ± 1.43
1-Undecanol	112-42-5	Alcohol	206.62 ± 3.94	14.5 ± 0.27	17.5 ± 0.15	10.35–11.54	82.5 ± 11.71
Crodatherm 17	n/a	Organic	184.74 ± 5.91	16 ± 0.04	18.6 ± 0.1	14.48–14.51	143.8 ± 13.44
Methyl hexadecanoate	112-39-0	Ester	205.03 ± 1.12	28.9 ± 0.05	31.5 ± 0.33	25.32–28.31	111.5 ± 10.32
CaCl_2_·6H_2_O	7774-34-7	Salt Hydrate	197.97 ± 6.22	29.2 ± 0.64	31.7 ± 0.73	−24.1–−16.4	n/a ^1^
Ethyl octadecanoate	111-61-5	Ester	188.74 ± 1.49	32.3 ± 0.08	34.5 ± 0	30.74–30.79	149.4 ± 17.28
CaBr_2_·6H_2_O	71626-99-8	Salt Hydrate	125.1 ± 2.78	32.7 ± 0.08	35.4 ± 0.09	23.37–24.46	46.1 ± 0.26
Eicosane	112-95-8	Paraffin	246.44 ± 5.02	36 ± 0.03	37.8 ± 0.06	35.94–35.96	154.5 ± 6.88
1-Tetradecanol	112-72-1	Alcohol	226.26 ± 2.47	37.3 ± 0.04	39.7 ± 0.1	37.66–37.77	110.8 ± 5.15
Methyl octadecanoate	112-61-8	Ester	229.16 ± 4.26	37.4 ± 0.03	40.1 ± 0.06	33.76–34.76	141 ± 4.06
Myristic acid-Stearic acid (50wt%/50wt%)	n/a	Organic Eutectic	179.97 ± 6.1	45.7 ± 0.07	48.2 ± 0.12	43.58–43.96	147.5 ± 1.33
1-Octadecanol	112-92-5	Alcohol	245.93 ± 1.09	56.8 ± 0.05	59.1 ± 0.07	57.19–57.26	147.6 ± 5.39
SAT	n/a	Salt Hydrate	228.28 ± 13.17	57.5 ± 0.11	60.4 ± 0.26	33.18–46.26	204.6 ± 1.87
(MgCl_2_-Mg(NO_3_)_2_)·6H_2_O (41wt%/59wt%)	n/a	Inorganic Eutectic	151.66 ± 0.12	58.1 ± 0.01	60.2 ± 0	31.85–32.89	60.5 ± 1.68
PEG 10000	25322-68-3	Organic	179.8 ± 3.03	59.3 ± 0.3	62.1 ± 0.12	46.52–50.14	364.8 ± 1.18
Hexadecanoic acid	57-10-3	Fatty Acid	216.72 ± 1.39	62.1 ± 0.01	63.7 ± 0.07	59.55–60.65	171.7 ± 2.68
Octadecanoic acid	57-11-4	Fatty Acid	228.26 ± 1.01	67.8 ± 0.2	70 ± 0.1	63.66–66.31	195.4 ± 1.4
meso-Erythritol	149-32-6	Sugar Alcohol	337.76 ± 7.51	118.7 ± 0.21	120.4 ± 0.17	22.99–32.29	192.3 ± 1.84
Dulcitol	608-66-2	Sugar Alcohol	352.91 ± 8.18	186.6 ± 0.51	189 ± 0.31	105.35–119.2	242.4 ± 1.57

1: n/a: not applicable.

## Supplementary Materials

The following are available online at https://www.mdpi.com/1996-1944/13/20/4486/s1, Figure S1: a) DSC 3+, b) DSC 823e, c) TGA/DSC 2, Figure S2: Three examples of prepared material for DSC measurement, Table S1: Enthalpy and onset temperature measured for Parafol 18-97 (octadecane) including their mean and standard deviation.
