# Supplementary material for: Consistent DSC and TGA Methodology as Basis for the Measurement and Comparison of Thermo-Physical Properties of Phase Change Materials
Source: Materials (Basel). 2020 Oct 10;13(20):4486. doi: 10.3390/ma13204486 (PMC7600577; doi:10.3390/ma13204486)
Supplement: Supplementary file 1 [file materials-13-04486-s001.pdf]

Supplementary Material

Consistent DSC and TGA methodology as basis for the measurement and comparison of thermo-physical properties of Phase Change Materials

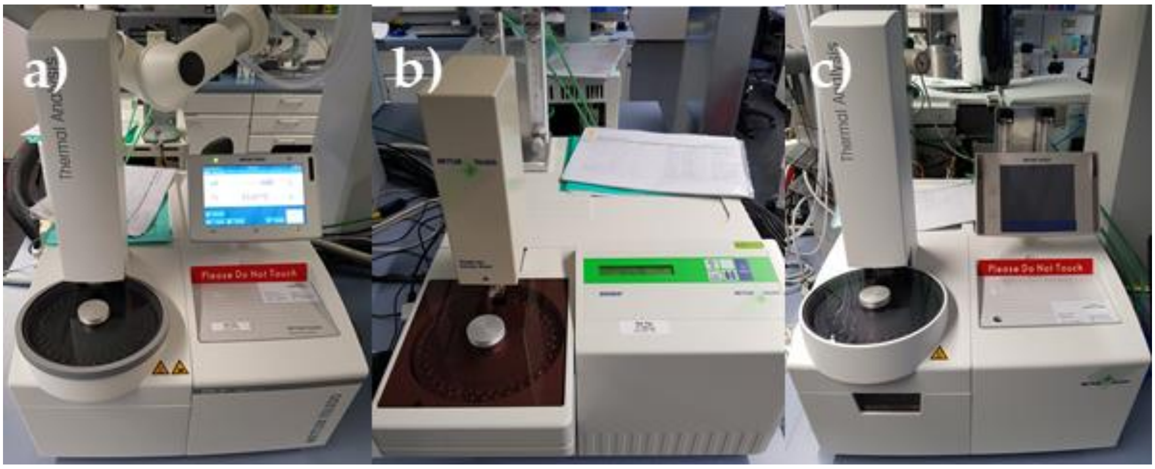

Figure S1. a) DSC 3+, **b**) DSC 823e, **c**) TGA/DSC 2.

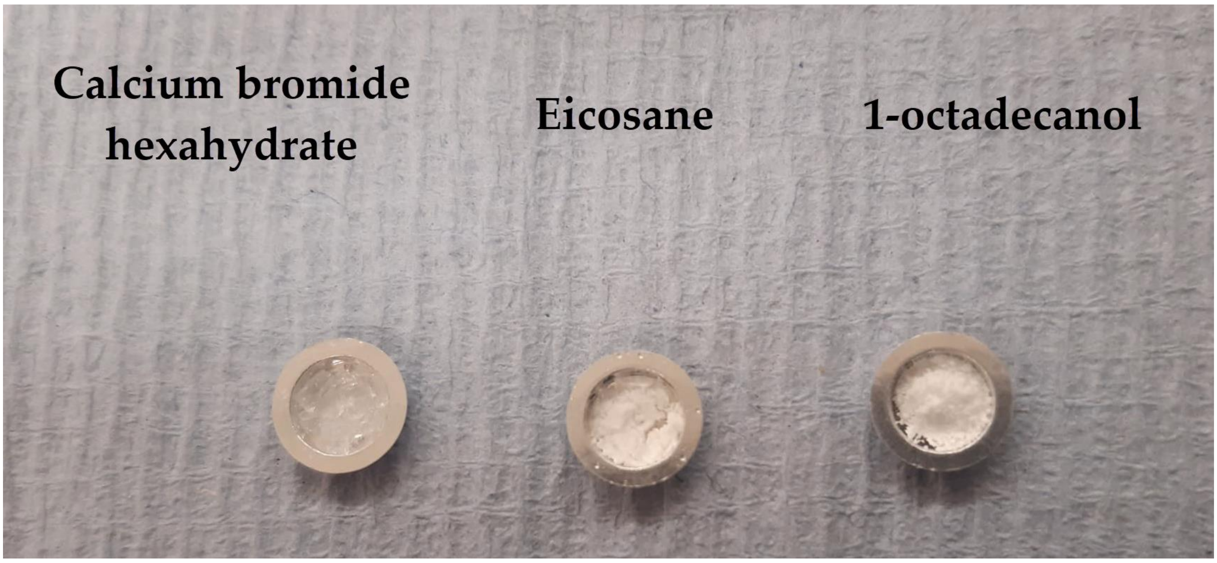

Figure S2. Three examples of prepared material for DSC measurement.

Table S1. Enthalpy and onset temperature measured for Parafol 18-97 (octadecane) including their mean and standard deviation.

Measurement number	Enthalpy [J g⁻¹]			Onset temperature [°C]		
	Measured	Mean	Standard Deviation	Measured	Mean	Standard deviation
1	224.82<br>224.69<br>224.73	224.75	0.07	27.29<br>27.31<br>27.52	27.37	0.13
2	221.67<br>221.09<br>221.08	221.28	0.34	27.3<br>27.27<br>27.26	27.28	0.02
3	225.61	225.72	0.09	27.53	27.53	0.02

225.76	27.51
225.78	27.55
